# Interfacial Charge Transfer Influences Thin-Film Polymorphism

**DOI:** 10.1021/acs.jpcc.1c09986

**Published:** 2022-02-01

**Authors:** Fabio Calcinelli, Andreas Jeindl, Lukas Hörmann, Simiam Ghan, Harald Oberhofer, Oliver T. Hofmann

**Affiliations:** † Institute of Solid State Physics, Graz University of Technology, 8010 Graz, Austria; ‡ Chair for Theoretical Chemistry and Catalysis Research Center, Technical University Munich, 85748 Garching, Germany; § Chair for Theoretical Physics VII and Bavarian Center for Battery Technology (BayBatt), University of Bayreuth, Universitätsstraße 30, 95447 Bayreuth, Germany

## Abstract

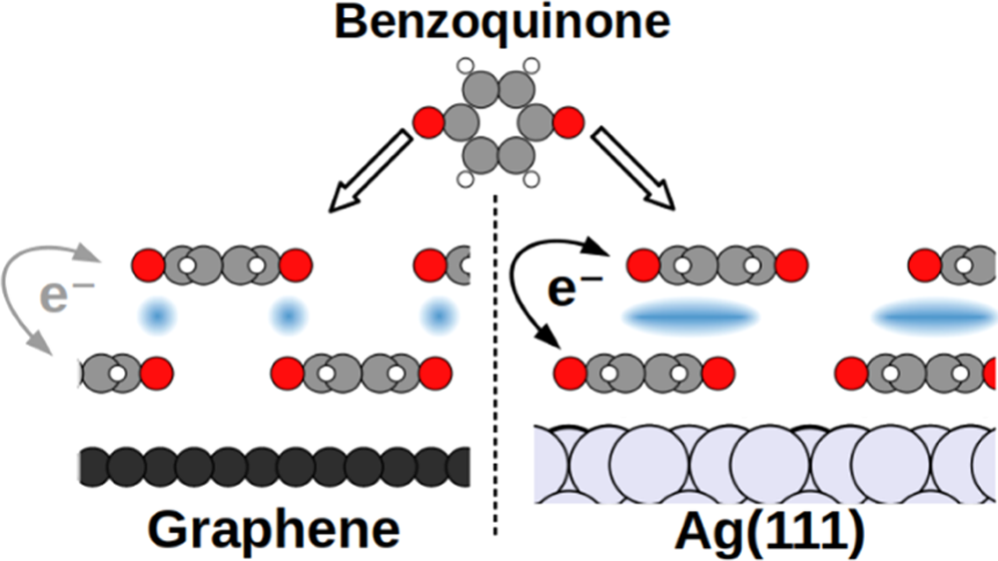

The structure and chemical composition are the key parameters influencing the properties of organic thin films deposited on inorganic substrates. Such films often display structures that substantially differ from the bulk, and the substrate has a relevant influence on their polymorphism. In this work, we illuminate the role of the substrate by studying its influence on *para*-benzoquinone on two different substrates, Ag(111) and graphene. We employ a combination of first-principles calculations and machine learning to identify the energetically most favorable structures on both substrates and study their electronic properties. Our results indicate that for the first layer, similar structures are favorable for both substrates. For the second layer, we find two significantly different structures. Interestingly, graphene favors the one with less, while Ag favors the one with more electronic coupling. We explain this switch in stability as an effect of the different charge transfer on the two substrates.

## Introduction

Organic thin films are materials of increasing interest, mainly by virtue of their application to the field of organic electronics. In comparison to inorganic alternatives, they present advantages such as mechanical flexibility and low cost. With a thickness ranging from less than a nanometer up to a few micrometers, organic thin films are commonly employed in the construction of organic field-effect transistors (OFETs),^1,2^ organic light-emitting diodes (OLEDs),^3^ and organic solar cells.^4^ Of particular interest are films composed of molecules that form ordered structures with relatively high charge carrier mobilities.^5−7^ In fact, the properties of molecular materials, and especially their charge carrier mobilities, depend drastically on the polymorph they assume, i.e., the relative arrangement of individual molecules in the thin film.^8,9^

Which polymorph a thin film forms depends not only on the fabrication conditions^10^ but also the nature of the substrate on which it grows has a decisive impact. Because the substrate interacts with molecules in the first layer and because it changes the way molecules interact with each other (e.g., because they become charged), the second and subsequent layers can either assume the same structure as the first,^11−13^ assume a bulk structure,^14^ or form a completely different structure altogether.^15^ The decisive role of the substrate is highlighted by reports, where even the same molecule forms different structures on different substrates.^16−18^

In this work, we shine light on the role of the substrate and tackle the question whether—and why—some substrates are more likely than others to induce polymorphs, which are beneficial for organic electronics. To this end, we use a combination of machine learning and first-principles calculations to investigate the structure of thin films of *para*-benzoquinone adsorbed on Ag(111) and on graphene.

## Computational Methods

To simulate the electronic structure of our systems, we performed density functional theory (DFT) calculations using the FHI-aims package,^72^ with the Perdew–Burke–Ernzerhof (PBE)^73^ exchange–correlation functional and the TS^surf^ correction^74,75^ for long-range dispersion interactions. The repeated slab approach was employed using a dipole correction^76^ to electrostatically decouple the periodic replicas in the *z* direction. Default “tight” basis sets were used for all chemical elements except Ag, for which a mixed quality numerical basis set (see ref (29) for details) was employed. A unit cell height of >80 Å was selected.

Predicting the structure of thin films is far from trivial. To date, a variety of specialized algorithms are available, which predict the structures of molecular crystals,^33−41^ their surfaces,^42^ and single molecules adsorbing on a surface^43−47^ or monolayers of molecules adsorbed on a substrate.^48−53^ Here, we use an extended version of the SAMPLE approach, which is specifically designed for inorganic/organic interfaces.^51^

When applying the SAMPLE approach, one starts with finding the local adsorption geometries that an isolated molecule could adopt on a surface. These structures act as building blocks for the subsequent structure search. To find all of these single-molecule local adsorption geometries on the surface, a three-step procedure is followed. First, a single molecule is relaxed at an arbitrary position on top of the substrate. Second, a Gaussian process regression tool equivalent to the BOSS approach^46^ is used to find all stationary points in the potential energy surface (PES) along three dimensions (translations along *X* and *Y*, rotation of the molecule around the axis perpendicular to the surface). Starting from these points, the adsorbate molecules are fully relaxed with the BFGS algorithm until the remaining forces on the atoms are below a threshold of 0.01 eV/Å. During this process, all substrate atoms are kept fixed. These optimized geometries later serve as building blocks.

As a second step in the SAMPLE approach, polymorph candidates—with numbers ranging in the millions—are built by assembling all possible combinations of the just obtained single-molecule building blocks in a variety of unit cells. A small subset of these polymorphs is then evaluated with DFT as described above. The resulting energies are used to train an energy model utilizing Bayesian linear regression. The trained energy model allows to predict the energies of all remaining polymorphs with a level of accuracy similar to the underlying electronic structure method. A more detailed explanation of the SAMPLE procedure is given in ref (51).

**Figure 1:**
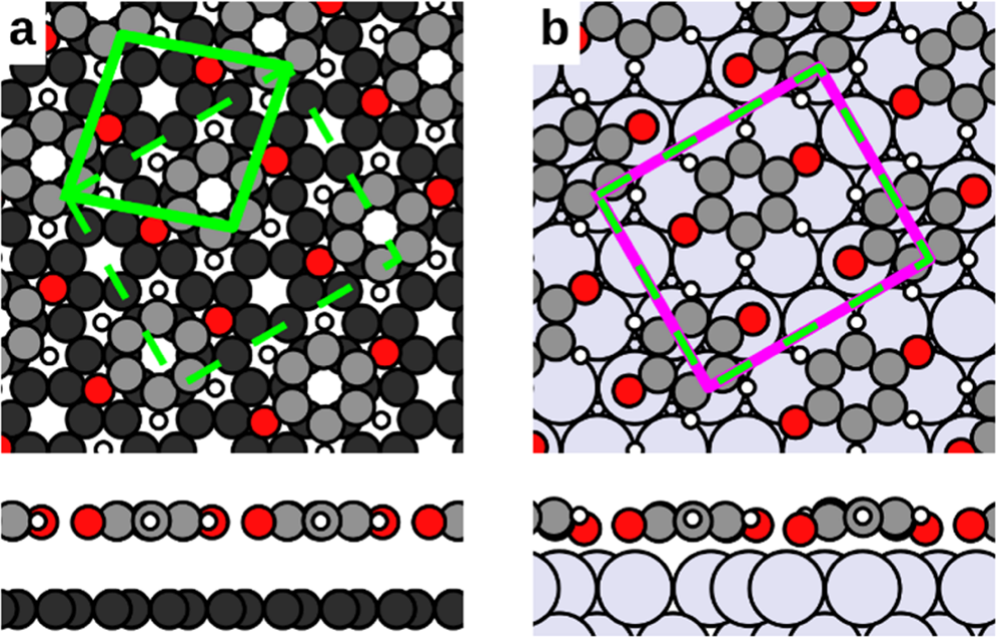
Geometry of the first layer of benzoquinone on (a) graphene and (b) Ag(111). The unit cell for benzoquinone on graphene is shown in solid green, and the unit cell for benzoquinone on Ag(111) is shown in purple. The dashed green lines indicate a unit cell equivalent to the unit cell on graphene but twice as large (its (1, 1, −1, 1) transform), which fits the Ag unit cell (purple) almost perfectly.

For Ag(111), the geometry of the first layer of benzoquinone was taken from an earlier work.^29^ The SAMPLE approach was applied to predict the structure of the second layer. As a substrate for the adsorption of the second layer, we used a geometry that includes Ag atoms and the first layer of benzoquinone. This geometry is shown in Figure 1b. To obtain the single-molecule building blocks, all calculations were executed on a 2 × 2 substrate cell, integrating in *k*-space on a grid of 3 × 3 points per primitive lattice direction and 1 *k*-point in the *Z* direction. To reduce the computational cost of running geometry optimizations with these systems, the search for local adsorption geometries was conducted on a gas-phase monolayer substrate, in which Ag atoms were removed. The adsorption energy of the adsorption geometries was evaluated, reintroducing the metal atoms for a single-point calculation. The full geometry, with metal atoms, was also used for all of the following stages of the structure search, which entailed working with polymorph candidates generated by assembling the building blocks. At this stage of the work, given the necessity to work with a wide variety of unit cells, the *k*-space integration was conducted on generalized Monkhorst–Pack grids.^78^ The grids were built with a sampling density of 11.74 Å, and this same value was used for all other systems as well. Among all polymorphs, a set of 250 was selected, employing the D-optimality criterion.^77^ Of these, 200 were randomly selected to compose a training set, while the remaining 50 were used as a test set. In addition, 961 “free-standing” calculations (i.e., polymorph candidates where the metal atoms and first-layer molecules were removed) were used to calculate priors for all intralayer interaction energies. After training with the conditions described before, SAMPLE predicted the adsorption energies of the test set with a root mean square error (RMSE) of 9 meV/nm^2^. Leave-one-out cross-validation (LOOCV)^79^ was also applied on the training set and gave an RMSE of 13 meV/nm^2^. We consider an accuracy of approximately 25 meV/molecule at room temperature (i.e., *k*_b_*T*), which, for the coverages found in our study, corresponds to about 60 meV/nm^2^, as an appropriate threshold to not miss important configurations.

For the graphene system, an analogous structure search procedure was applied separately to both the first and the second layers of benzoquinone. For the first layer, the search for local adsorption geometries was conducted on a 5 × 5 graphene cell. This cell size ensured that the interaction of the benzoquinone molecule with its periodic replicas was negligible. At this stage, the *k*-space integration was conducted on a grid of 6 × 6 points per primitive lattice direction and 1 *k*-point in the *Z* direction. For the SAMPLE prediction, 100 calculations were used, 60 as a training set and 40 as a test set, together with 1000 “free-standing” calculations for the intralayer prior. At this stage, the *k*-space integration was conducted on generalized Monkhorst–Pack grids.^78^ The prediction resulted in an RMSE of 8 meV/nm^2^ on the test set and a LOOCV-RMSE of 16 meV/nm^2^.

For the second layer, the structure shown in Figure 1a was set as a substrate primitive unit cell. The search for local adsorption geometries was conducted on a 2 × 2 substrate cell, and the *k*-space integration was conducted on a grid of 6 × 6 points. For the SAMPLE prediction, 250 calculations were used, 200 as a training set and 50 as a test set, together with 997 calculations on hypothetical, gas-phase layers for interaction priors. At this stage, the *k*-space integration was conducted on generalized Monkhorst–Pack grids.^78^ The prediction resulted in an RMSE of 24 meV/nm^2^ on the test set and a LOOCV-RMSE of 47 meV/nm^2^. Further details about the results of the structure search procedure can be found in the Supporting Information to this paper.

To compare the effect of the two different substrates on the electronic structure of the first adsorbate layer, we performed calculations of the adsorption-induced charge rearrangement Δρ, which is defined as
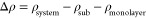
1where ρ_system_, ρ_sub_, and ρ_monolayer_ are the charge densities of the combined system, of the substrate, and of the isolated benzoquinone monolayer, respectively. From this quantity, we can derive an estimate of the net charge transfer from below the substrate to above the substrate by estimating the maximum value of
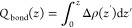
2To compare the energetics of different molecular arrangements, we obtained the pure electrostatic interaction between molecules (see Figure 5b) by applying an energy decomposition scheme. This decomposition scheme combines the electron densities of the isolated fragments to calculate the classical electrostatic energy.^65,67−69^ These calculations were performed with a code designed for periodic systems (see ref (65) for details). To emulate a cluster system, the molecules were placed in a 25 × 25 × 50 Å unit cell. Additional charge was added with a layer of point charges analogously to the CREST method.^80^

To obtain an estimate of the charge carrier mobility for different systems, we performed calculations of electronic coupling terms between molecules and layers with the Löwdin-orthogonalized^81^ second version of the projection-operator diabatization method POD2L,^71^ which was recently demonstrated to yield very accurate results for organic molecules.^62^ For these calculations, FHI-aims default light basis sets were used in place of the tight basis sets, as the former were found to be more numerically stable under the required block-diagonalization scheme. In the case of molecular dimers, the coupling between lowest unoccupied molecular orbitals (LUMOs) of the two molecules was calculated. In the case of bilayers on graphene, in which a unit cell of each layer contains one molecule, the coupling between the LUMOs of the two isolated monolayers at the Γ point was calculated. In the case of bilayers on Ag(111), a unit cell of each layer contains two molecules; as a consequence, for each layer, the molecular LUMOs combine to form two orbitals, LUMO and LUMO + 1 of the isolated monolayer, which are almost perfectly degenerate in energy. Interlayer couplings were computed calculating the couplings between all 4 possible combinations of orbitals (LUMO–LUMO, LUMO–LUMO + 1, LUMO + 1-LUMO, LUMO + 1-LUMO + 1), summing the 4 values and dividing by 2 to obtain per-molecule values directly comparable to those on graphene.

## Results and Discussion

Both Ag and graphene are sensible electrode materials in organic electronics.^19−21^ At the same time, they show fundamentally different interactions with organic molecules: Ag is a weakly reactive substrate, which readily undergoes charge-transfer reactions and can form weak covalent bonds with organic adsorbates.^22−29^ Conversely, graphene hardly forms covalent bonds at all. Benzoquinone was chosen as a model molecule due to its small size (reducing the computational cost) while exhibiting π-conjugation and functionalization with carbonyl groups. As we have previously shown, the intermolecular interactions of this molecule are qualitatively similar to those of technologically more relevant, larger analogues like 5,12-pentacenequinone.^29−32^

Before considering thin-film growth, it is necessary to look at the structure that the first layer of benzoquinone forms on the two substrates. For Ag(111), the polymorph candidates have been obtained in an earlier work,^29^ while for graphene, a structure search is performed anew through the SAMPLE approach (see Computational Methods for details about the approach). The best polymorph in the SAMPLE ranking on graphene has one molecule per unit cell and is presented in Figure 1a. In this geometry, molecules adsorb at a height of approximately 3.3 Å and remain almost perfectly flat.

For benzoquinone on Ag(111), we find a comparable structure among the energetically best polymorph candidates (details in the Supporting Information). This configuration is shown in Figure 1b. Its unit cell contains two molecules, placed on a top site and on a bridge site of the metal surface. The molecules adsorb at a height of about 2.6 Å and are slightly bent, with oxygen closer to the metal substrate than the carbon backbone.

The two geometries appear strikingly similar, and in fact, an equivalent cell of the graphene monolayer, with twice the area, is virtually identical (deviations lower than 0.01 Å) to the cell of the monolayer on Ag(111) (dashed green cell and purple cell in Figure 1b). The fact that the first layer on both substrates shows equivalent lattice parameters and molecular alignment means that any subsequent layers will be subjected to identical stress, and to equivalent templating effects from the first layer. In other words, we can expect that any differences in the energetics and structure of the second layer stem directly from the (electronic) influence of the substrate.

As a first step in describing thin films, we study the second molecular layer, and we invoke two assumptions. First, we assume that the geometry of the first layer only undergoes minor changes when the additional material is deposited—in particular, the unit cell remains fixed. We note that, in practice, this is not always the case, as in some systems the first layer reorients to form a more tightly packed layer.^54−56^ However, predicting such reorientations is beyond the scope of the present work. Second, we assume Frank–van Der Merwe growth, i.e., each layer does not start forming until the previous layer is full. This assumption is reasonable here because benzoquinone shows strongly attractive intermolecular interactions. Together, these two assumptions allow us to use the SAMPLE approach. For this, we employ the monolayer geometries of benzoquinone (plus metal/graphene) as effective substrate unit cells and search for and combine the local adsorption geometries in the second layer. To obtain accurate energies, after the ranking of the polymorph candidates by SAMPLE, we perform full geometry optimizations for the 10 best structures to allow the molecules in the second layer to assume more favorable orientations toward the first layer. For these optimizations, molecules in the first layer were also allowed to relax; the top 2 layers of Ag were kept free, allowing the surface to partially reconstruct, while the bottom 6 layers were kept fixed; all graphene atoms were kept fixed since initial tests showed that the graphene substrate relaxed by less than 0.01 Å/atom (with an energy variation of less than 10 meV/nm^2^).

**Figure 2:**
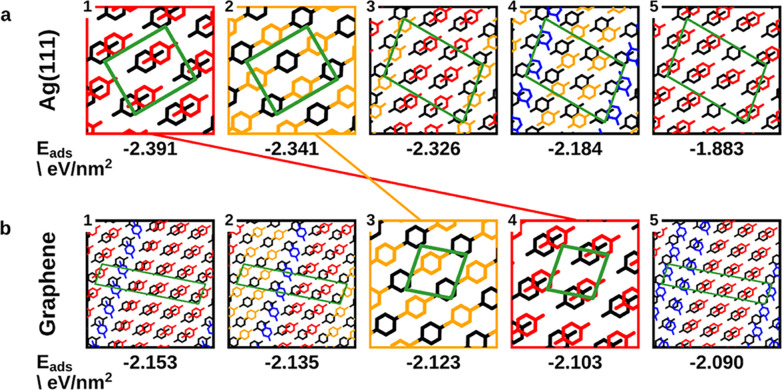
DFT adsorption energy and graphical representation of the five best configurations of the bilayer of benzoquinone on (a) Ag(111) and (b) graphene. The boxes corresponding to molecule-on-gap and molecule-on-molecule (for explanation, see main text) are colored in orange and red, respectively. In the geometry representations, Ag and graphene are omitted, the first layer of adsorbates is colored in black, and the second layer is colored according to the adsorption positions (i.e., similar positions have the same color).

For Ag, the five energetically best bilayer structures are shown in Figure 2a. The ranking is performed according to energy per area, the most sensible measure for the stability of close-packed adsorbate polymorphs.^57^ In the energetically most favorable structure, the benzoquinone molecules in the first and the second layer are partly on top of each other, with one (negatively charged) oxygen of one molecule always aligned with the center (i.e., the least negative region) of the ring of a molecule in the other layer. We refer to this alignment, which is shown in Figure 2a by red molecules in the top layer, as molecule-on-molecule (MoM) hereafter. The second-best geometry is already 50 meV/nm^2^ worse in energy. In this geometry, the molecules in the second layer are located above “gaps” of the first layer (marked in orange in Figure 2a). Only the carbonyl groups of the first and the second layer are on top of each other, with oppositely directed dipoles, presumably leading to electrostatic attraction. To distinguish this alignment from the others, we refer to it as molecule-on-gap (MoG) hereafter. The energetically next-higher lying structures are combinations of MoM and MoG, variations thereof, and structures with lower coverages.

On graphene, we also find the MoM and the MoG geometry as energetically favorable structures. However, in salient contrast to the situation on Ag, here, the MoG structure is energetically more beneficial than MoM by 20 meV/nm^2^. Only two structures are found that are energetically even better than MoG and MoM. Both of these structures are noticeably more complex than MoG and MoM, featuring five adsorbates per unit cell and several adsorption positions similar to MoM and MoG. For the sake of conciseness and clarity, we will focus the following discussion on the MoM and the MoG structures only. A brief discussion of structures 1 and 2 can be found in the Supporting Information.

Since the charge carrier mobility (or, more precisely, the electronic coupling) of a crystal depends on the wave function overlap,^8,58,59^ already a visual inspection of the MoM and MoG geometries lets us expect that this property will be very different for the two geometries. The fact that the ordering of the two polymorphs reverses depending on the substrate, therefore, deserves further scrutiny, and we should attempt to explain the reasons for this switch and its consequences on interlayer electronic coupling.

When considering only the second layer, on each substrate, the MoM and MoG polymorphs exhibit the same unit cell vectors and very similar geometries, differing mostly by a translation relative to the first benzoquinone layer. Thus, we expect the switch in the energetic ordering to be caused by a variation in the interlayer interactions between the first and the second layer.

**Figure 3:**
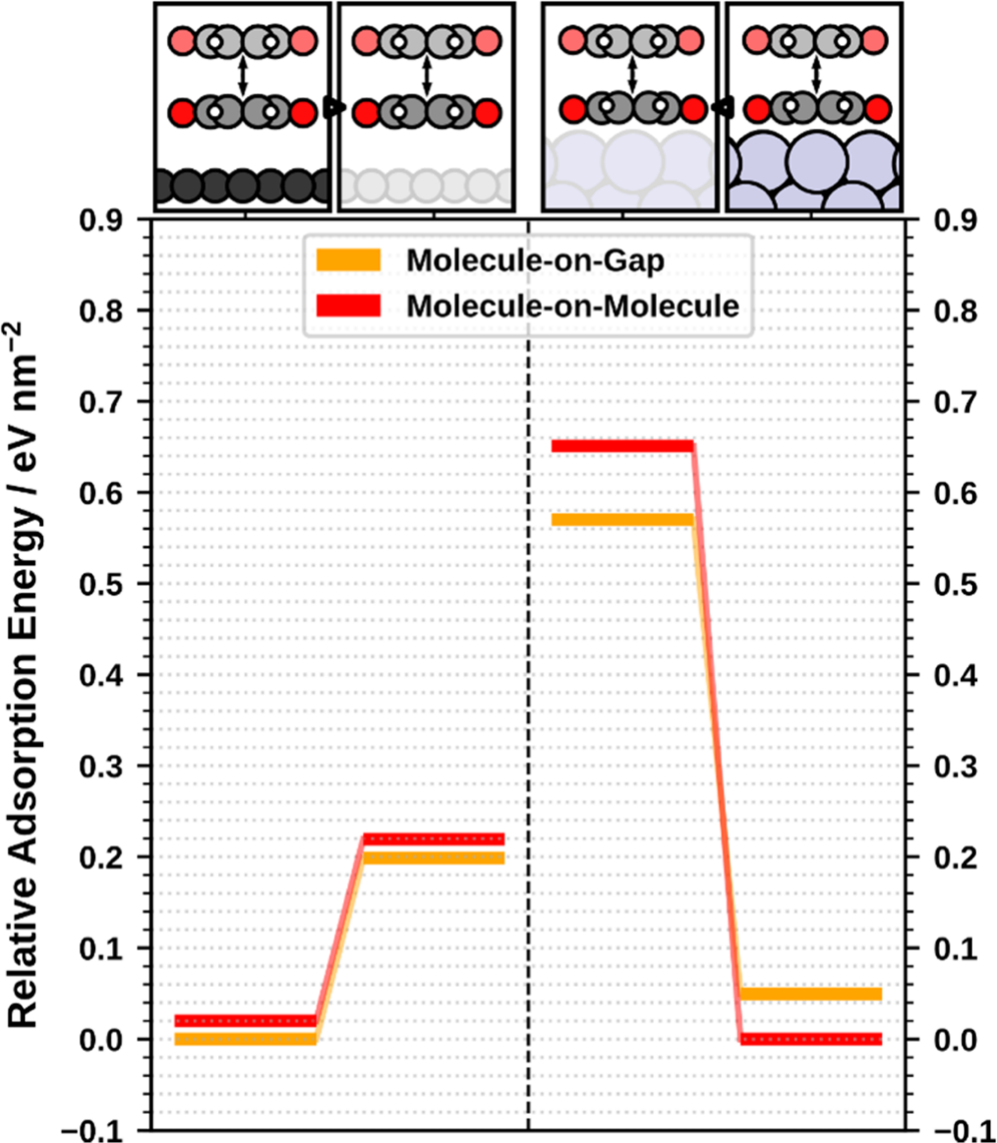
Adsorption energies of MoM and MoG adsorbing on the two monolayer-on-substrate geometries (left: graphene; right: Ag(111)) and on gas-phase monolayers having the same geometry as the adsorbed monolayers but with no substrate atoms. The energies are given relative to the value of the most stable geometry for each full-substrate system.

To verify that the switch in stability is caused directly by the different substrates, and not by the small geometric differences in the first layer, we examine the variation in adsorption energy that occurs if we keep the geometry of the first layer fixed but remove all graphene or Ag atoms (Figure 3). We find that for the case of graphene, MoM and MoG experience destabilizations that are moderate and fundamentally equivalent, i.e., a graphene substrate does not notably affect the energetic ordering. For Ag(111), when removing the substrate, MoM becomes energetically destabilized with respect to the MoG geometry. This indicates a stronger influence of the substrate on the MoM structure compared to MoG. We can thus conclude that the Ag substrate massively changes the way the first and the second layer interact with each other. Specifically, we find that the Ag substrate significantly stabilizes the MoM geometry, explaining why it is favored on Ag but not on graphene.

**Figure 4:**
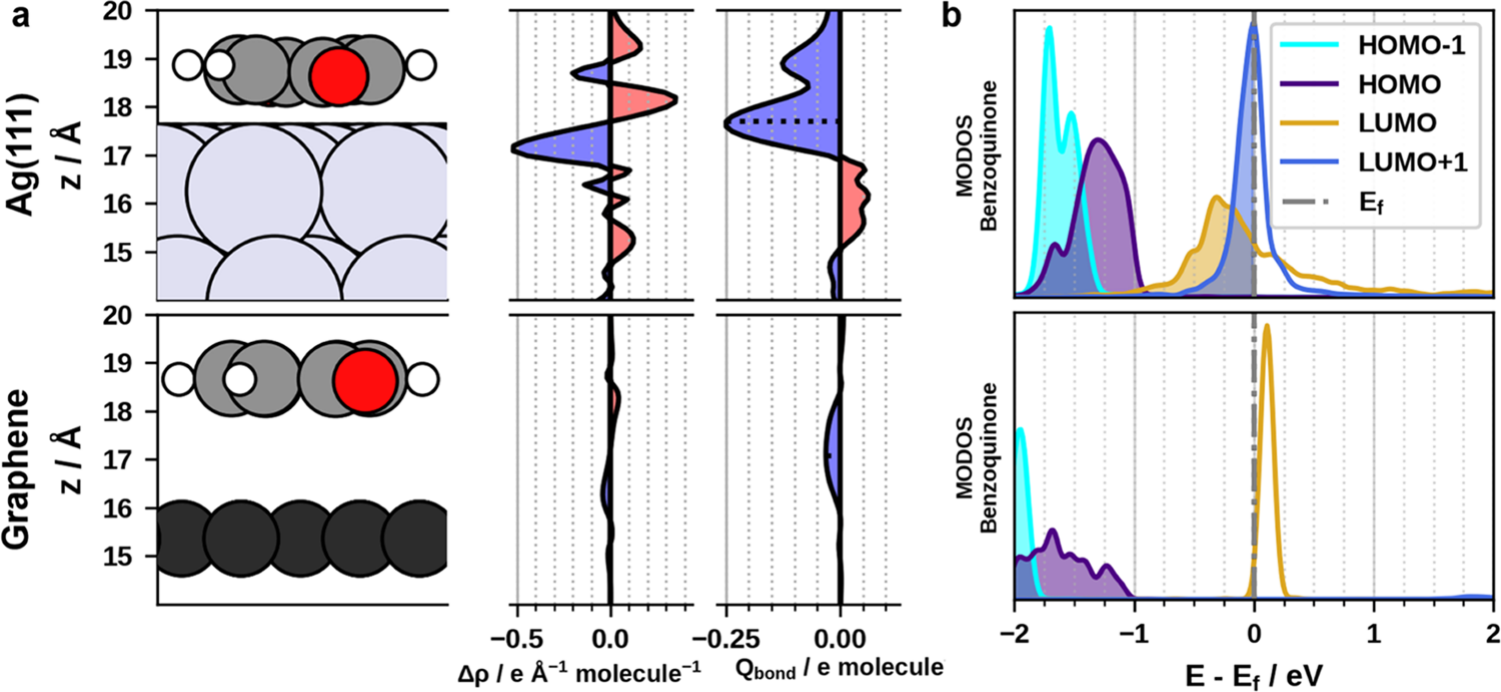
(a) Analysis of the charge transfer between Ag(111)/graphene and the first layer of benzoquinone, with Δρ being the variation of net charge, averaged on a *xy* plane, due to adsorption, and *Q*_bond_ being the integral of Δρ from the bottom to each *z* value. (b) Molecular-orbital projected density of states (MODOS) analysis showing the contributions of the molecular orbitals of the isolated benzoquinone molecule to the density of states of the combined system.

We now need to ask which underlying mechanism stabilizes the MoM geometries. We can trace the effect back to the charge rearrangements resulting from the contact between the substrate and a molecular layer. To illustrate this, we calculated the adsorption-induced charge rearrangements Δρ and the net charge transfer max(*Q*_bond_) for the benzoquinone monolayers on Ag and on graphene (for details, see Computational Methods). The profiles of Δρ and *Q*_bond_ along the *z* axis are shown in Figure 4a and lead to a value of max(*Q*_bond_) of −0.249 for benzoquinone on Ag(111) and −0.031 for benzoquinone on graphene.

In other words, for Ag, over the area occupied by one benzoquinone molecule, a charge corresponding to 0.25 electrons is transferred from below the substrate surface to above it. Conversely, graphene is practically inert, and the electron transfer is negligible. Furthermore, by conducting a comparative molecular-orbital projected density of states (MODOS) analysis,^60,61^ detailed in Figure 4b, we find that the LUMO and LUMO + 1 of the benzoquinone monolayer (corresponding to the LUMOs of the two benzoquinone molecules in the unit cell) fall largely under the Fermi energy for Ag(111) but remain above it for graphene. As a consequence, the LUMO of the benzoquinone layer gets filled in the case of Ag, reaching an occupation of 1.25 electrons, while in the case of graphene, it remains substantially empty at an occupation of 0.05 electrons.

**Figure 5:**
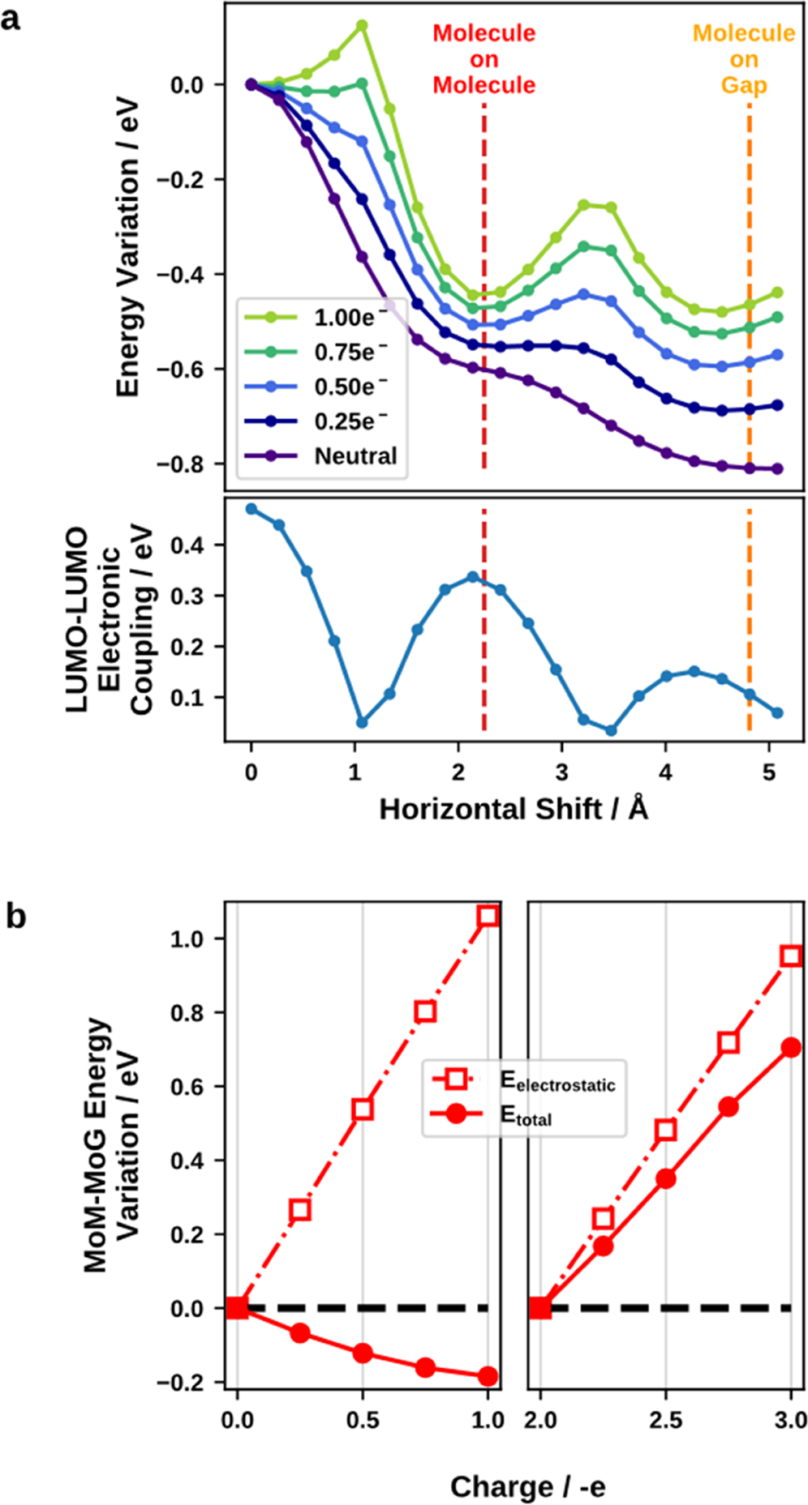
(a) Variation of the total energy (without van der Waals interactions) and LUMO–LUMO electronic coupling for different shifts along the main molecular axis of a benzoquinone dimer. The shifts corresponding to the MoM and MoG structures are indicated with vertical lines. (b) Variation of the energy difference between MoM and MoG as a function of extra charge. Here, in addition to the values of charge used in panel (a), we see the effect of charges in the 2–3 e^–^ range. In this range, additional charge occupies the antibonding orbital combination.

This different charge transfer directly impacts the interaction with the second molecular layer. To analyze the effect of extra charge on interlayer interactions, we use a simple dimer model composed of two stacked benzoquinone molecules. The two molecules are arranged at a distance of 3 Å along the *z* direction, which is a reasonable approximation of the interlayer distances for our systems. They are then shifted with respect to one another along the long molecular axis. The shifting starts from a position of congruence in *x*–*y* coordinates and includes positions corresponding to both the MoM and MoG offsets. For each position, the electronic energy of the system (i.e., the total energy without van der Waals contributions) is evaluated together with the coupling between the LUMOs of the two molecules (Figure 5a) obtained with the methodology described in ref (62). The suitability of this model for describing the interactions of the full monolayers is discussed in the Supporting Information.

It has been observed that, in analogous cases, one can find an inverse correlation between stability and the highest occupied molecular orbital (HOMO)–HOMO coupling, as a consequence of Pauli repulsion.^63−65^ In our case, as we are interested in the response of the system to the introduction of additional electronic charge, we focus on the coupling between LUMOs. For the neutral system (shown in purple), there is no correlation between the coupling and the energy. This also would not be expected since the orbitals are completely empty. Rather, the energy of the system decreases systematically as the molecules are shifted away from each other. This can be attributed to a reduction in Pauli pushback, as the wave functions no longer overlap.

The situation changes notably when additional charge is introduced. As can be seen, particularly for larger charges, the energy profile now shows an inverse correlation with the LUMO–LUMO coupling, i.e., situations with a large coupling are energetically more favorable than those with a small coupling. The MoM geometry has a significantly larger coupling than the MoG geometry (although both are local maxima) and is therefore more stabilized (up until a charge of two electrons, see below). This is in accordance with what we have observed in the behavior of the configurations in Figure 3.

This behavior can be readily rationalized by valence-bond theory. When two identical molecules come in contact, their LUMOs (originally at the same energy) will hybridize and form a bonding and an antibonding linear combination. The splitting depends on the orbital coupling,^66^ i.e., the bonding combination is more strongly bonding the larger the coupling is. If the system is neutral, this has no effect on the total energy. However, when electrons are introduced, they will first occupy the bonding linear combination. As long as there are less than two additional electrons per dimer, only the bonding one will be occupied, resulting in a net energy gain that is larger the larger the coupling is. Conversely, when more than two electrons are introduced, the effect reverses. This tendency is confirmed by Figure 5b, where the variation of the MoM–MoG energy differences is plotted as a function of charge. One can observe that MoM is favored when increasing charge between 0 and 1 electrons but is disfavored when increasing charge between 2 and 3 electrons. For each value of additional charge, a term describing the pure electrostatic interaction between layers has been calculated. One can see that this electrostatic term disfavors MoM for all values of additional charge, proving that the stabilization of MoM in the 0–1 electron range is caused by the previously discussed orbital hybridization, and not by purely electrostatic effects.

In other words, we have demonstrated that the charging of the first layer on Ag(111) is the main factor governing the preferability of MoM compared to MoG because the additional charge in the first layer directly benefits geometries with a large LUMO–LUMO overlap.

This provides a simple and solid explanation of why the two arrangements present different stabilities on the two substrates. In addition, it provides an important hint toward the consequences of this stability switch: it is known that charge carrier mobility, within the model of the hopping regime, is fundamentally influenced by the coupling between the origin and destination orbitals.^59^ Generally, our results indicate that substrates that undergo significant charge transfer with the first layer will facilitate the formation of polymorphs that have a large LUMO–LUMO overlap. Because the LUMO–LUMO coupling is a relevant ingredient for the electron mobilities of the compound,^8,70^ it stands to reason that these polymorphs generally exhibit superior properties. In our case, we can estimate the rate of interlayer charge transfer for the two systems by calculating the electronic coupling between LUMO orbitals with the projection-operator diabatization method.^62,71^ The results are shown in Table 1.

**Table 1:** Interlayer Electronic Couplings for MoM and MoG Structures

electronic coupling (eV/molecule)	molecule-on-molecule	molecule-on-gap
molecular dimer	0.320	0.106
bilayer on graphene	0.269	0.209
bilayer on Ag(111)	0.372	0.277

One can see that MoM exhibits superior electronic coupling over MoG for all of the systems we consider. For the single molecular dimer from Figure 5, the difference is very large, and although a part of this difference is due to the nature of the dimer model, which, lacking periodic boundary conditions, presents some intrinsic geometric differences from the full monolayer geometries, the trend is persistent for more complex systems, up to and including the full bilayer geometries found by our structure search.

This shows that the influence of the choice of the substrate is crucial for the performance of any device and exemplifies that, even when we can examine the fortuitous case in which two different substrates would seem to induce the same geometry to the first layer, the influence beyond the first layer can be enough to drastically alter the geometry and, thus, the properties of the system.

## Conclusions

We have studied the structure of the first two layers of benzoquinone on two different substrates. Employing first-principles calculations in combination with machine learning, we have found that for the first layer, similar structures are favorable for both substrates. For the second layer, two structures are very favorable for both systems, but their ranking is swapped for the two substrates. This difference in ranking is a consequence of the difference in the LUMO–LUMO coupling for the two different structures in the second layer. Hereby, the MoM structure has a large coupling compared to the MoG structure. Without induced charge, MoG is energetically more favorable compared to MoM. When charge is induced into the first molecular layer (as is the case for Ag), MoM becomes energetically stabilized due to the LUMO–LUMO coupling. This points to the fact that the two different structures induced by the two substrates would exhibit different vertical charge carrier mobilities. Our computational study therefore indicates that substrates which undergo a notable charge transfer with the first layer are more likely to induce polymorphs with large(r) electronic coupling and, hence, charge carrier mobilities.

## Supporting Information Available

Details on the convergence of *k*-space integration grids; graphical representation of all local adsorption geometries; details about the SAMPLE prediction of the second layer on Ag(111) and of the first and second layer on graphene, including geometry optimizations; discussion of the best first-layer polymorphs to be chosen as a substrate for the second layer; further discussion of the best configurations for the second layer of benzoquinone on graphene; and comparison of the dimer model to more complex models for the comparison of MoM and MoG electronic coupling (PDF)
