# Supplementary material for: Interfacial Charge Transfer Influences Thin-Film Polymorphism
Source: J Phys Chem C Nanomater Interfaces. 2022 Feb 1;126(5):2868–76. doi: 10.1021/acs.jpcc.1c09986 (PMC8842301; doi:10.1021/acs.jpcc.1c09986)
Supplement: Supplementary file 1 — jp1c09986_si_001.pdf [file jp1c09986_si_001.pdf]

Supporting Information to “Interfacial Charge Transfer Influences Thin-Film Polymorphism”

*Fabio Calcinelli[1], Andreas Jeindl[1], Lukas Hörmann[1], Simiam Ghan[2], Harald Oberhofer[2] and Oliver T. Hofmann[1]**

[1]Institute of Solid State Physics, Graz University of Technology, 8010 Graz, Austria

[2]Chair for Theoretical Chemistry and Catalysis Research Center, Technical University Munich, 85748 Garching, Germany

*Corresponding author: **o.hofmann@tugraz.at**

Contents

1. Convergence of k-grids 2

2. Application of the SAMPLE approach for finding and ranking polymorphs 4

3. Selection of the first-layer polymorphs to be used as substrates for the growth of the second layer 13

4. Discussion of complex second-layer configurations of benzoquinone on graphene 14

5. Comparison of models of increasing complexity for reproducing the LUMO-LUMO overlap of different configurations 15

6. References 16

1. Convergence of k-grids

BQ on Ag(111) 2nd layer

For the calculation of local adsorption geometries, a grid of 6x6 k-points was used. Given that the calculations were run on a 2x2 cell, this corresponds to a 12x12 k-grid on the 1st layer unit cell. As the 1st layer unit cell has a surface corresponding to 12 Ag unit cells, arranged as a 3x4 grid, this value corresponds to a grid of 36x48 points, which is in excess compared to the value used in the reference.[1]

For the calculations following the application of SAMPLE, automatically generated generalized Monkhorst-Pack (GMP) grids were used.[2] The density parameter (corresponding to the reciprocal of the maximum distance between k-points in reciprocal space) was converged to 11.74 Å, giving an error of less than 10 meV in adsorption energy for the most stable local adsorption geometry, as shown in Figure S1.

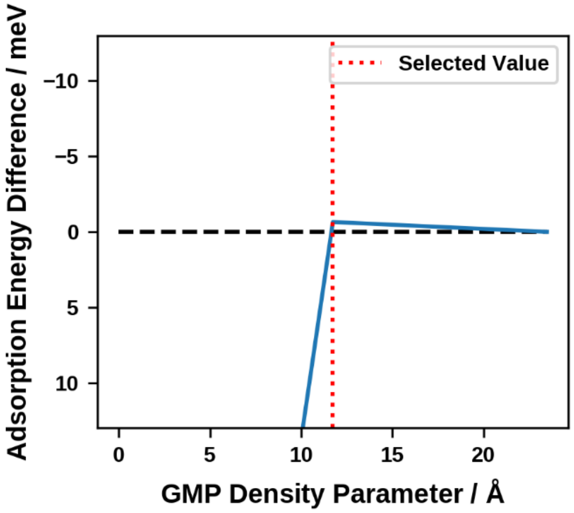

Figure S1. *Convergence of adsorption energy with respect to GMP density parameter for the second layer of benzoquinone on Ag(111). The energy difference is computed with respect to the densest grid.*

BQ on graphene 1st layer

For the calculation of local adsorption geometries, a grid of 30x30 k-points for a graphene primitive unit cell was selected (see Figure S2) and adapted to the size of each supercell.
For the calculations following the application of SAMPLE, automatically generated generalized Monkhorst-Pack grids were used. The density parameter was converged to 11.74 Å, giving an error of less than 10 meV in adsorption energy for the most stable local adsorption geometry, as shown in Figure S3.

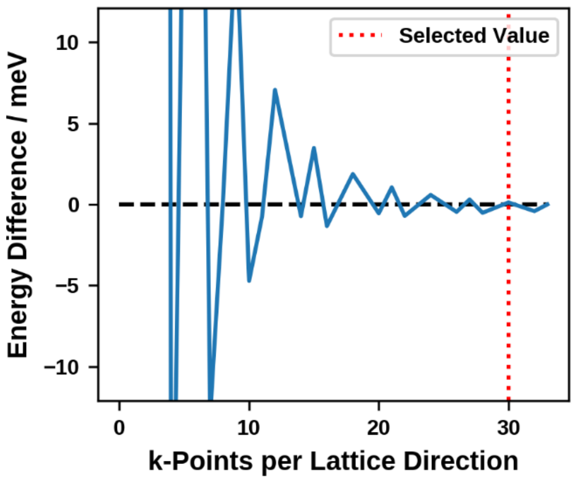

Figure S2. *Convergence of total energy with respect to number of k-Points for graphene. The energy difference is given with respect to the densest grid.*

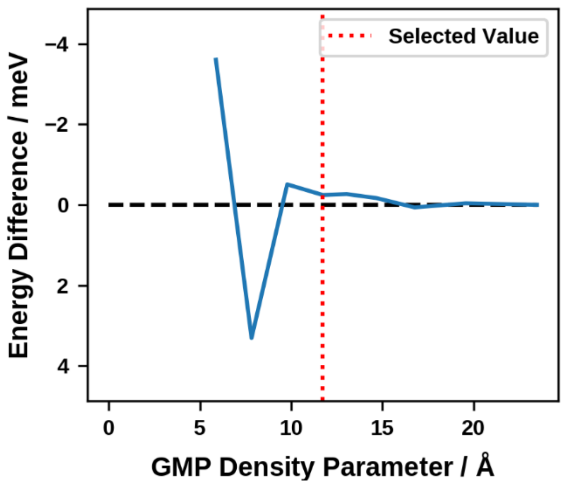

Figure S3. *Convergence of adsorption energy with respect to GMP density parameter for benzoquinone on graphene. The energy difference is computed with respect to an overconverged system with 180x180 k-Points for primitive graphene unit cell.*

BQ on graphene 2nd layer

For the calculation of local adsorption geometries, a grid of 7x7 k-points was used. Given that the calculations were run on a 2x2 cell, this corresponds to a 14x14 k-grid on the substrate primitive unit cell. As the substrate primitive unit cell has a surface corresponding to 8 graphene primitive unit cell, this value is in excess compared to the value used for the 1st layer of BQ on graphene.

For the calculations following the application of SAMPLE, automatically generated generalized Monkhorst-Pack grids were used. The density parameter was converged as shown in Figure S4, and a value of 11.74 Å (the same used for the previous layer) was chosen for simplicity.

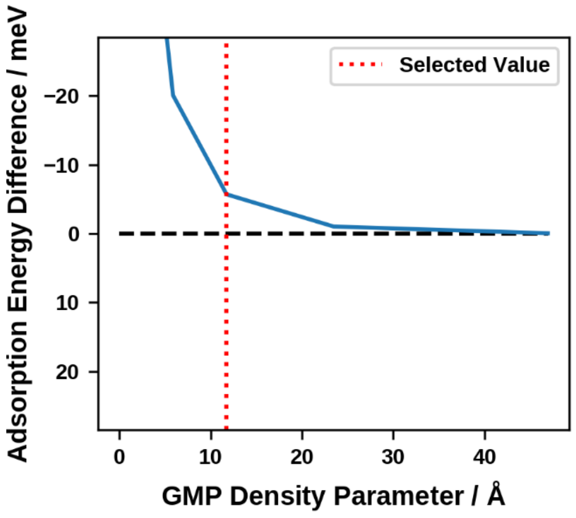

Figure S4. *Convergence of adsorption energy with respect to GMP density parameter for the first layer of BQ on graphene. The energy difference is given with respect to the densest grid.*

2. Application of the SAMPLE approach for finding and ranking polymorphs

We use SAMPLE to find and rank all possible polymorphs for the systems we consider. In this section we include not only the application of the SAMPLE algorithm, but also the preliminary stage of finding local adsorption geometries (building blocks) and the geometry optimizations performed on the best structures.

Finding local adsorption geometries (building blocks)

As reported in the main text, the following procedure was applied to find local adsorption geometries (the building blocks for all configurations): first, a single molecule was relaxed at an arbitrary position on top of the substrate unit cell to find a suitable adsorption height; secondly, the BOSS approach[3] was employed to find all stationary points in the PES along three dimensions (translations along X and Y, rotation of the molecule around the axis perpendicular to the surface); finally, all the geometries corresponding to these points were relaxed while keeping all substrate atoms fixed. If two or more optimizations led to the same position, the redundant ones were eliminated. All local adsorption geometries for the 1st and 2nd layer of benzoquinone on graphene are reported in Figures S5 and S6, together with the relative adsorption energies.

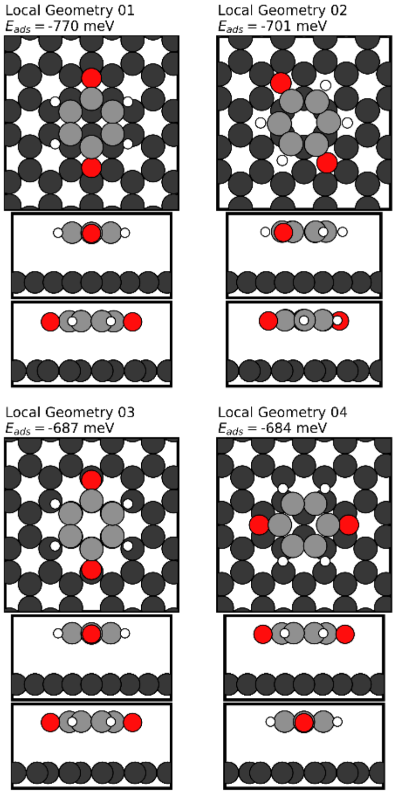

Figure S5. *Local adsorption geometries for the 1st layer of benzoquinone on graphene (top view and two orthogonal side views).*

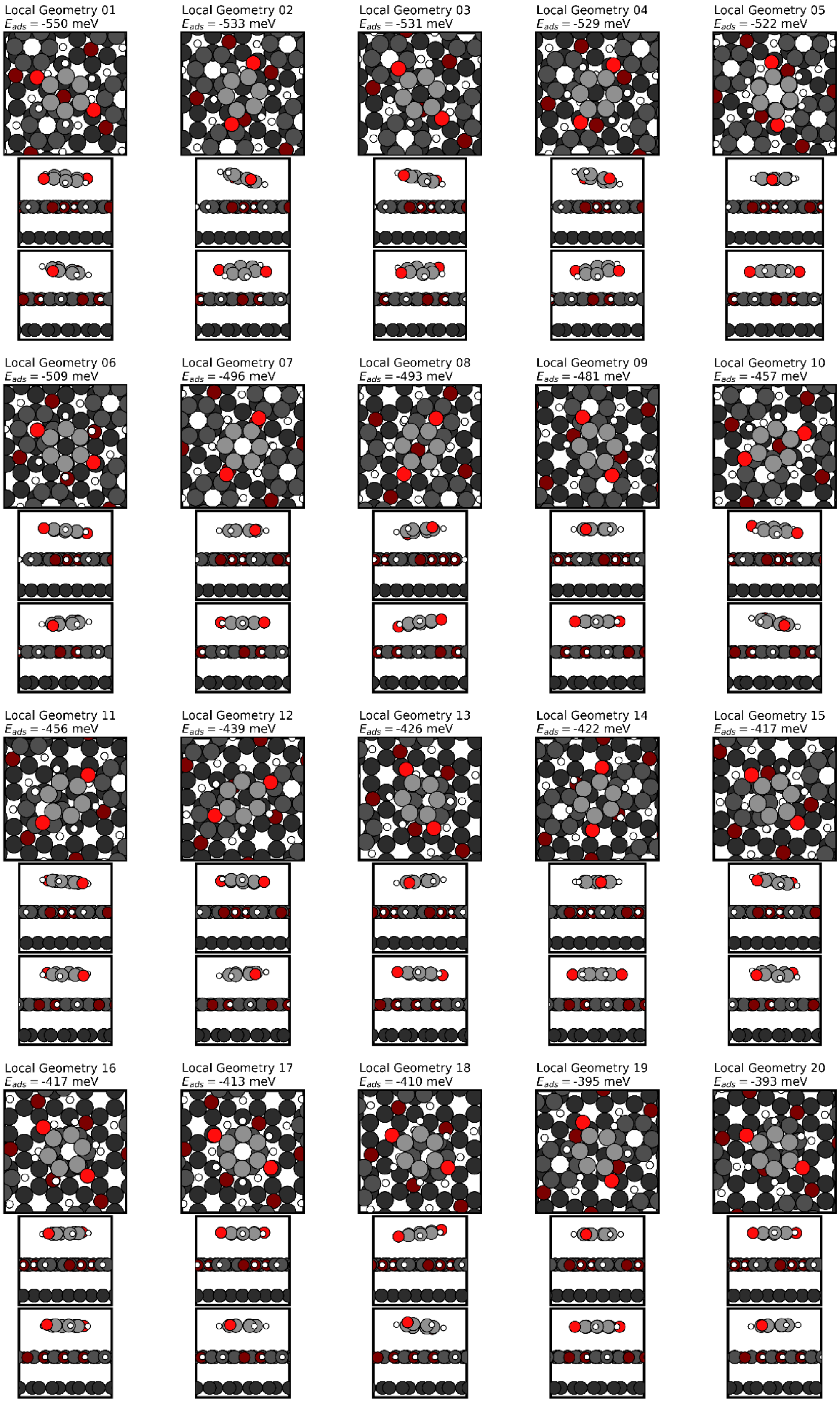

Figure S6. *Local adsorption geometries for the 2nd layer of benzoquinone on graphene (top view and two orthogonal side views).*

In the case of benzoquinone on Ag(111), local adsorption geometries for the second layer were found on a free-standing benzoquinone substrate, and the resulting geometries were then recalculated on the full metal substrate. All local adsorption geometries on the free-standing benzoquinone substrate are reported in Figure S7, while the variations in energy and ordering that result from the reintroduction of the Ag atoms are indicated in Table S1.

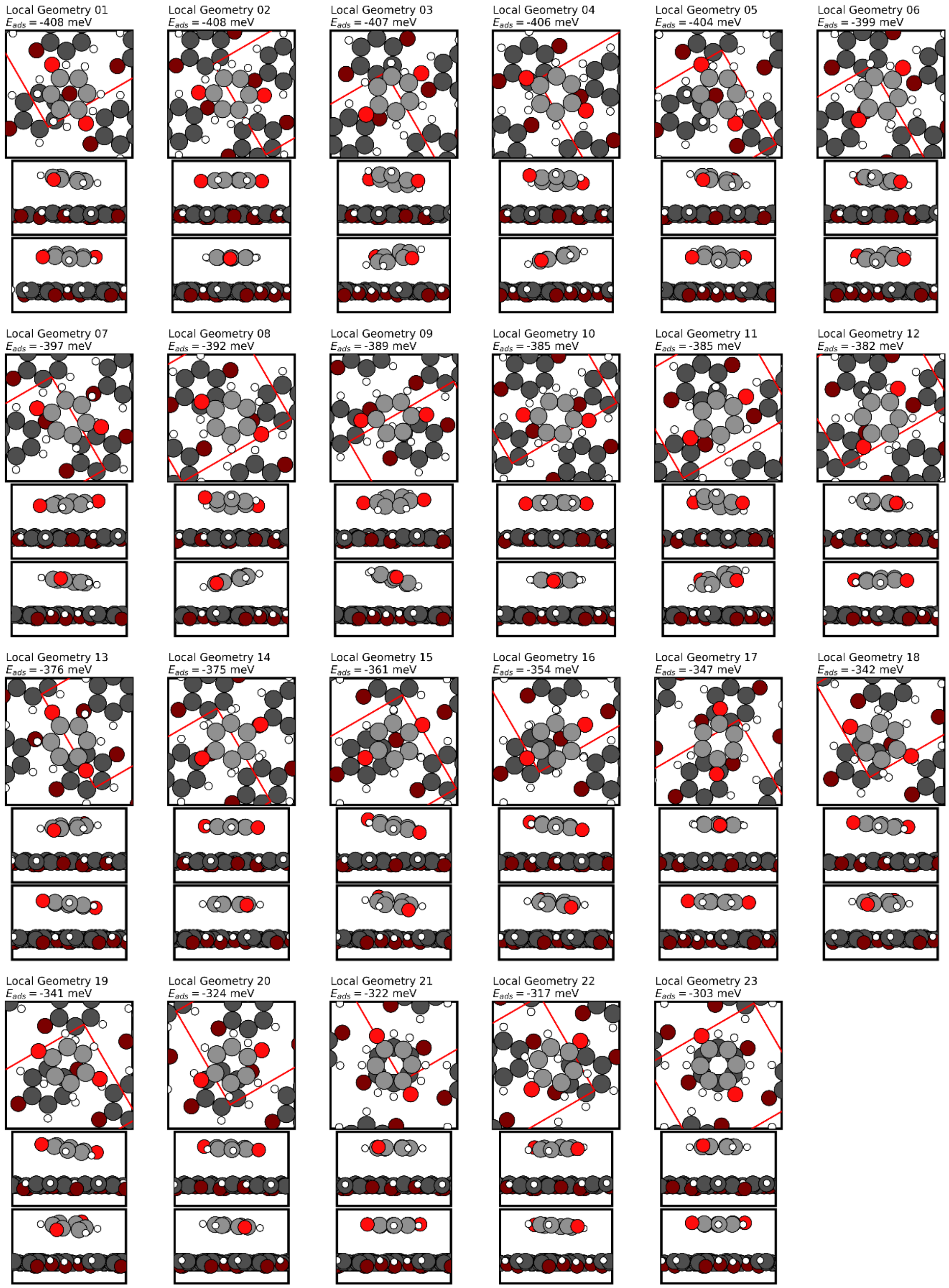

Figure S7. *Local adsorption geometries for the second layer of benzoquinone on Ag(111), as found on a free-standing monolayer of benzoquinone (top view and two orthogonal side views). The unit cell of the substrate is shown in red, allowing to distinguish the two non-equivalent molecules of the substrate.*

Table S1. *Adsorption energies of all local adsorption geometries of the 2nd layer of benzoquinone on graphene and original ranking and adsorption energy on the simplified free-standing-monolayer substrate.*

local adsorption geometry	***adsorption energy (meV)***	***original ranking on free-standing monolayer substrate***	***original adsorption energy on free-standing monolayer substrate (meV)***
1	-483	2	-408
2	-478	3	-407
3	-477	4	-406
4	-476	15	-361
5	-473	11	-385
6	-472	16	-354
7	-468	6	-399
8	-464	13	-376
9	-464	10	-385
10	-463	7	-397
11	-462	12	-382
12	-459	8	-392
13	-455	14	-375
14	-448	9	-389
15	-436	1	-408
16	-430	5	-404
17	-404	18	-342
18	-397	19	-341
19	-379	20	-324
20	-373	17	-347
21	-373	22	-317
22	-333	21	-322
23	-271	23	-303

Generating configurations with SAMPLE

SAMPLE produces a wide variety of configuration by producing a set of unit cells, and trying all combinations of local adsorption geometries and their symmetric equivalents that can be fit in each cell.[4] When executing this step of the SAMPLE approach, one must decide which cells to build, and how many molecules to try and fit in them. By using a big maximum cell size and a high maximum number of molecules, one would allow for the prediction of a larger number of configurations. This would of course allow for the possibility of finding new structures, but it must be noticed that the number of resulting configurations gets out of hand very rapidly, so one should always limit these parameters in order to obtain a reasonable number of structures. Moreover, one should modulate the maximum number of molecules to the size of the unit cell, to avoid producing a large number of mostly useless configurations with very low coverages. In addition to this, one must establish the distance threshold under which molecules are considered to be colliding, and the configuration containing them is discarded. This is defined for each possible combination of chemical elements, and must be chosen so that no configuration with strongly repulsive interactions is produced. The values of all these parameters, for the different systems on which SAMPLE was applied, are reported in Table S2, together with the number of unit cells and configurations that were produced.

Table S2. *Parameters for the construction of configurations by SAMPLE and resulting numbers of cells and configurations.*

	**2nd layer benzoquinone on Ag(111)**	**1st layer benzoquinone on graphene**	**2nd layer benzoquinone on graphene**
**cell areas (n. of primitive unit cells)**	1 - 3	5 - 66	1 - 6
**number of molecules**	Area 1: 1 - 3<br>Area 2: 3 - 5<br>Area 3: 3 - 7	Area 5 – 29: 1 – 3<br>Area 30 – 31: 3 – 4<br>Area 32 – 66: 3	Area 1: 1 - 2<br>Area 2: 1 - 4<br>Area 3: 2 - 6<br>Area 4: 2 - 8<br>Area 5: 3 - 10<br>Area 6: 4 - 9
**distance thresholds (Angstrom)**	HH: 1.500<br>OH: 1.500<br>OO: 2.200<br>CH: 2.000<br>CO: 2.400<br>CC: 3.000	HH: 1.337<br>OH: 1.441<br>OO: 2.127	HH: 1.500<br>OH: 1.500<br>OO: 2.200<br>CH: 2.000<br>CO: 2.400<br>CC: 3.000
**number of generated cells**	7	1087	22
**number of generated configurations**	83,044	26,518,330	349,483

Training and evaluation of the energy model

The energy model of SAMPLE is trained for each system on a D-optimally selected set of configurations. A fraction of the selected configuration set is used as validation set to estimate the performance of the model. In addition, a leave-one-out-cross-validation (LOOCV) approach is followed to gain an additional indication of the accuracy of the prediction. A graphical comparison between the exact and predicted values of adsorption energies for the three systems is provided in Figure S8, while the results of the performance evaluation are summarized in Table S3.

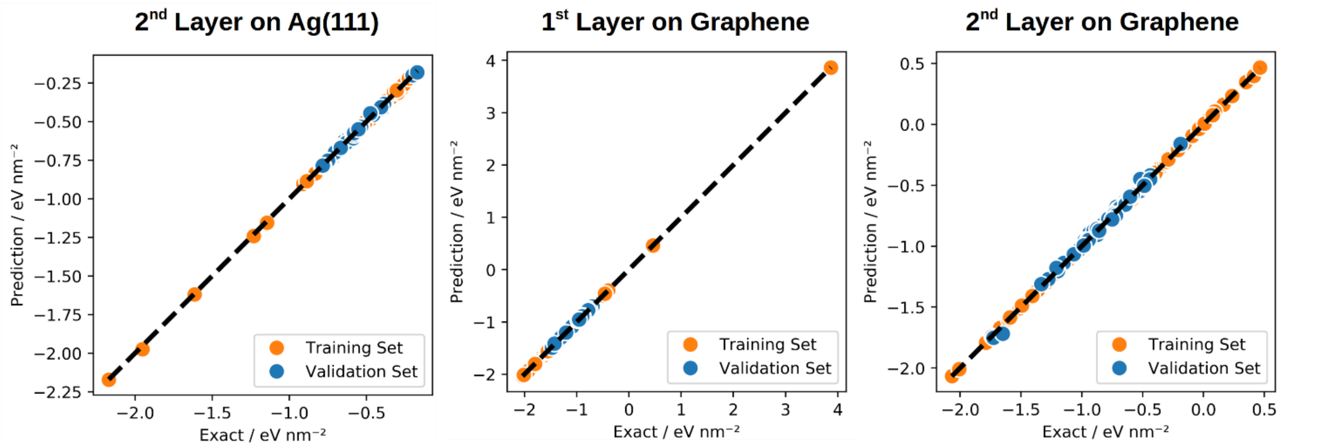

Figure S8. *Parity plots (prediction vs exact) for the three systems on which the SAMPLE approach was applied.*

	on validation set		LOOCV	
	RMSE (meV/nm^2)	max error	RMSE	max error
2nd layer on Ag(111)	8.7	28.8	13.3	53.6
1st layer on graphene	7.8	17.7	15.7	79.5
2nd layer on graphene	24.2	72.1	47.3	339.5

Table S3*. Performance of the energy model for the three considered systems: the root mean square error and the maximum error are provided for the evaluation on the validation set and for the leave-one-out-cross-validation procedure.*

Rankings of all configurations

SAMPLE gives access to a prediction of the adsorption energies of all configurations. This allows to rank the configurations from the most stable to the least stable. In Figure S9 the rankings for all three SAMPLE runs are shown.

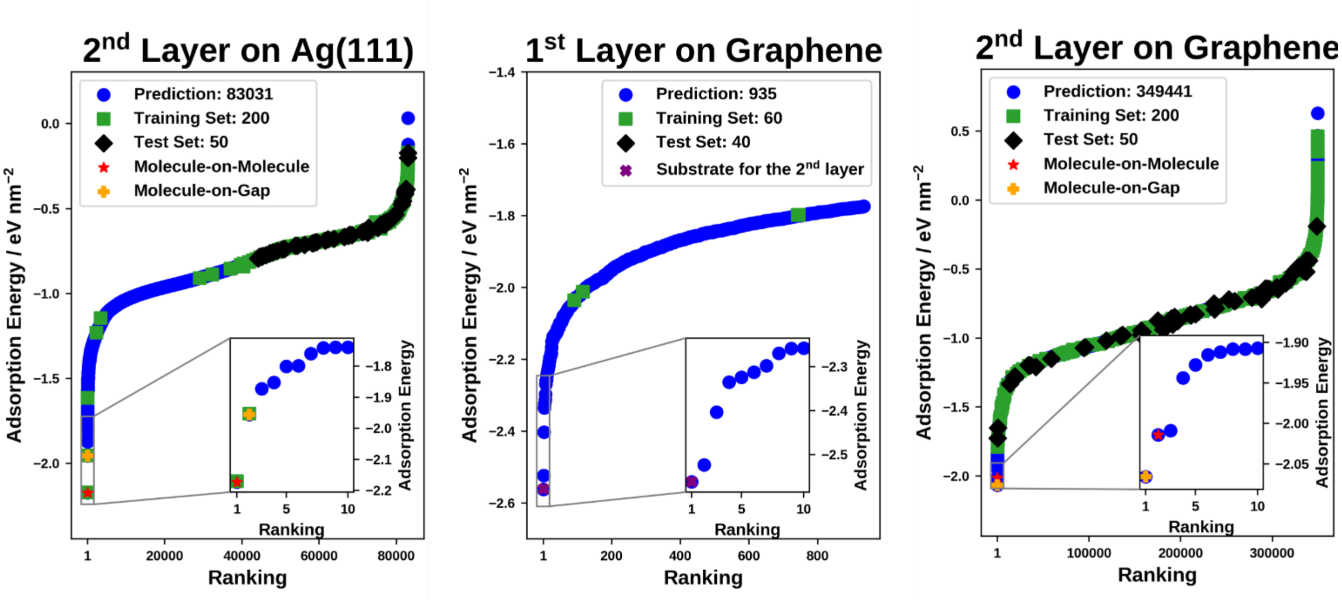

Figure S9. *Ranking of all configurations for all three systems. For the 1st layer of graphene, given the extremely large number of configurations, only a subset is shown. This is obtained by taking the best 1000 configurations and filtering out any configurations with reducible unit cells. The same duplicate removal procedure has also been applied to the top 1000 configurations of the other two systems, and this explains why the total number of plotted configurations is slightly smaller than the total number of constructed configurations reported in Table S2.*

Geometry optimization of the best configurations

Once SAMPLE has allowed us to select the most stable configurations according to its energy model, the DFT energies for the 10 best configurations of each structure are calculated. In addition, geometry optimizations are run allowing the newly formed layer of adsorbates to relax. Subsequently, also the first layer and – in the case of Ag(111) – the top layers of substrate are allowed to relax. This is necessary in the case of second-layer prediction, as the corrugated adsorption surface of the first layer combined with the generally weaker adsorption energies makes it easier for the adsorbates to rearrange compared to their single-molecule adsorption geometry. A summary of these optimizations for the second layer of benzoquinone on Ag(111) is shown in Figure S10.

One can notice a very good agreement between the SAMPLE prediction and the single-point DFT calculations. On the other hand, the relaxation of the second layer of adsorbates produces big changes in energy, that fortunately do not change the ordering of the structures, except for structures 3 and 4. Structures 5-10 are very similar, and are fundamentally variations of structure 1 with some defects. On a positive note, one can notice that the re-relaxation of the 1st layer produces a small and uniform variation in energy, showing that the second layer of adsorbates does not influence the first layer strongly.

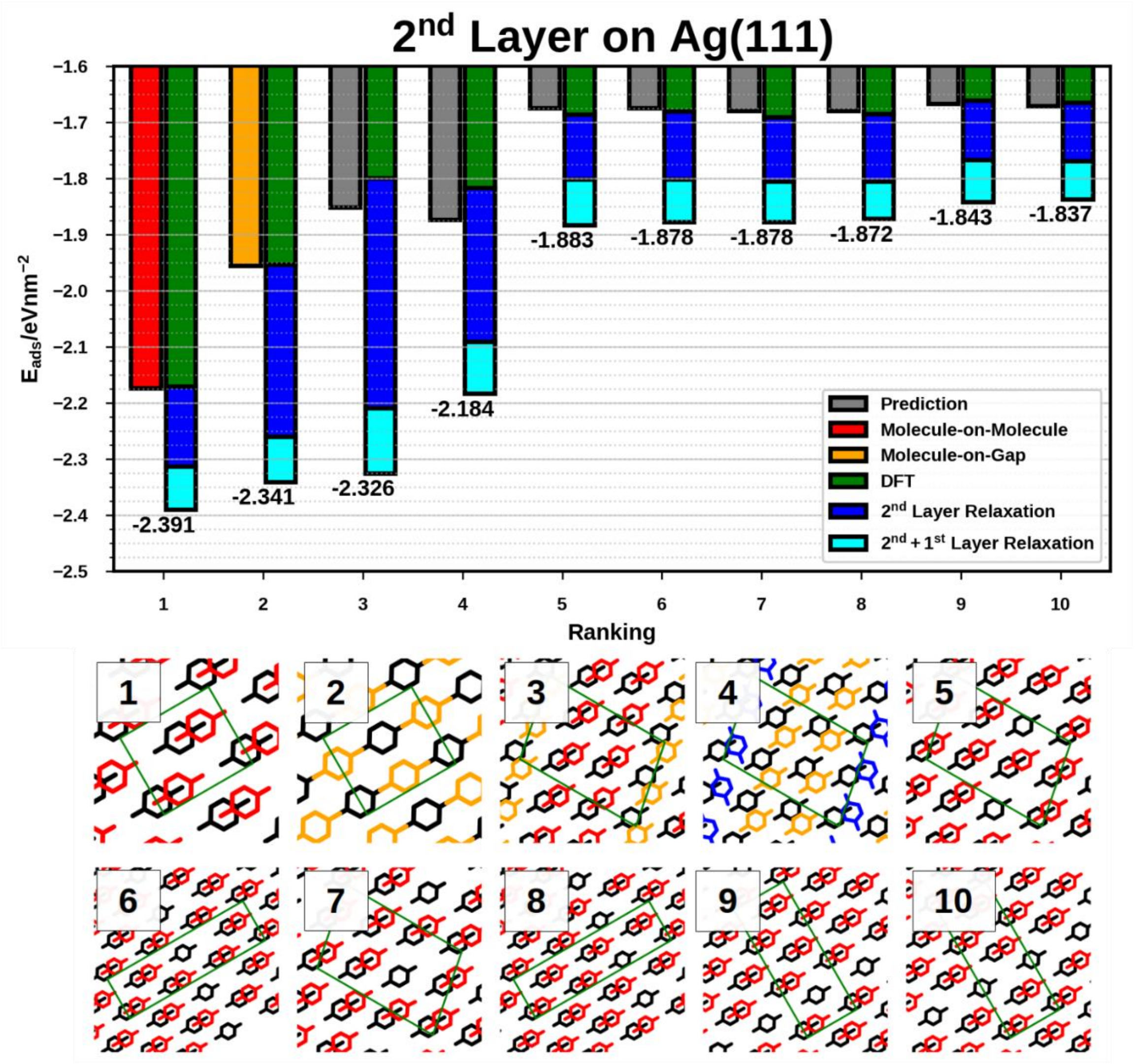

Figure S10. *Energies from predictions, single-point DFT calculations, and DFT geometry optimizations of the 10 best configurations of the 2nd layer of benzoquinone on Ag(111) and graphical representation of all 10 configurations.*

A summary of optimization results for graphene is given in Figure S11. For this system, while the agreement between SAMPLE predictions and DFT data remains good, the geometry optimizations produce intense and irregular changes in energies, which result in several switches in ordering going from the prediction results to the post-optimization results. The relaxation of the first layer also produces stronger perturbations compared to Ag(111), as a consequence of the weaker interaction between substrate and 1st layer. In particular, we can see a set of structures with a very elongated unit cell arriving to the top 5 positions of the ranking. Of these, structures 1a to 1c are fundamentally equivalent, so 1b and 1c are discarded. Structures 3a, 3b and 3d are also basically identical to the single-cell configuration 3c and are therefore discarded. This gives us the top-5 ranking shown in Figure 2.

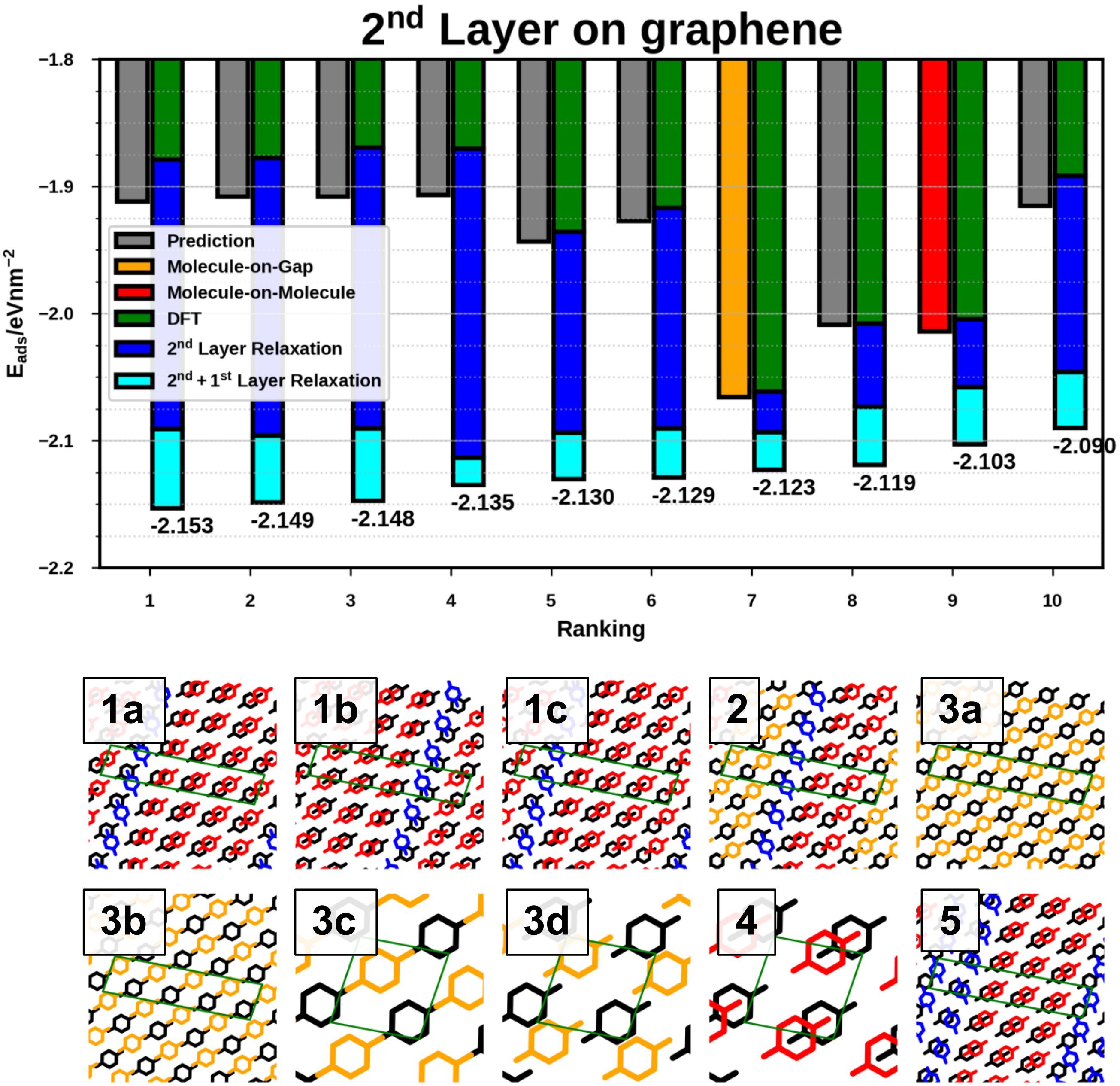

Figure S11. *Energies from predictions, single-point DFT calculations, and DFT geometry optimizations of the 10 best configurations of the 2nd layer of benzoquinone on graphene and graphical representation of all 10 configurations.*

3. Selection of the first-layer polymorphs to be used as substrates for the growth of the second layer

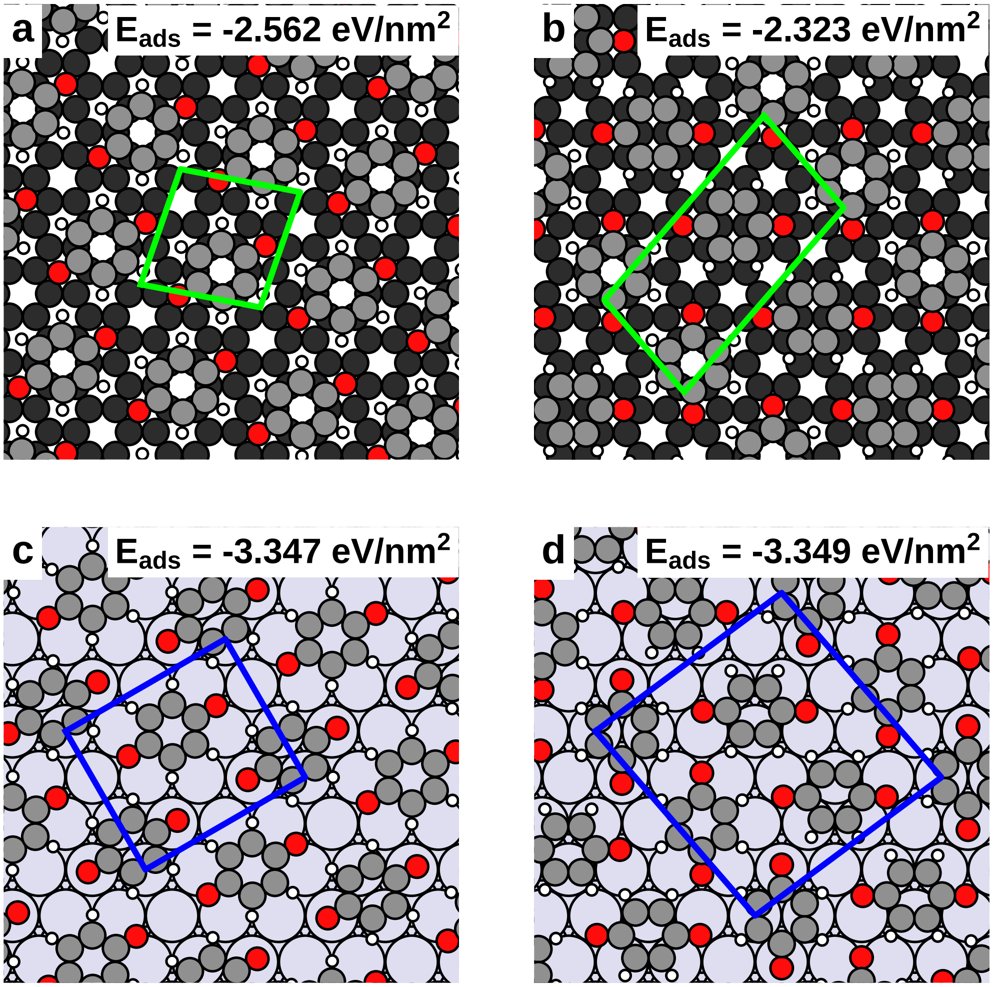

Figure S12. *1st (a, selected as substrate for the second layer) and 5th (b) configuration of benzoquinone on graphene, according to adsorption energy per area. 2nd (c, selected as substrate for the second layer) and 1st (d) best configuration of benzoquinone on Ag(111).*

Applying the SAMPLE approach to the prediction of the second molecular layer requires choosing a first-layer polymorph as substrate. The most sensible criteria for the selection of the most stable closed-packed polymorph is the energy per area.[5] In the case of benzoquinone on graphene the most stable polymorph is the one we have selected as substrate for the second layer, with an adsorption energy per area of -2.562 eV/nm^2. It is shown in Figure S12a. This polymorph is also the best in terms of adsorption energy per molecule. The second best polymorph is an extremely similar structure, with the same molecular arrangement slightly shifted with respect to the substrate. After these two polymorphs, all other structures are less stable by more than 100 meV/nm^2. In the case of benzoquinone on Ag(111), a polymorph with an identical structure to the best structure on graphene is found, with an energy that is tied to the energetically best polymorphs within our prediction uncertainty (-3.347 eV/nm^2 versus -3.349 eV/nm^2). This structure, which we show in Figure S12c, is the structure we have selected for the comparison of the two substrates.

4. Discussion of complex second-layer configurations of benzoquinone on graphene

As shown in Figure 2 and Figure S11, the 2nd layer of benzoquinone on graphene presented a few configurations more stable than Molecule on Molecule and Molecule on Gap, namely configurations 1 and 2 in Figure 2. While we have focused our discussion on simpler configurations, MoM and MoG, it is useful to discuss the properties of these more complex configurations. In particular, it should be understood what makes them so favorable, and why they appear for graphene and not for Ag(111).

First, it should be noticed that these structures share a fundamental feature: they are all constituted of a 5x1 unit cell, in which 4 molecules are aligned like MoM and MoG while the 5th molecule is rotated by 90°. The 4 aligned molecules are placed on the first molecular layer in positions similar to those of MoM and MoG, but each molecule is in a slightly shifted position, with respect to the first layer, compared to the previous one. The pattern is identical for configurations 1 and 2, the only difference being a small difference in alignment with respect to the first molecular layer. Consequently, we will now focus on configuration 1 to explain the cause of its stability, and the results will also apply to configuration 2.

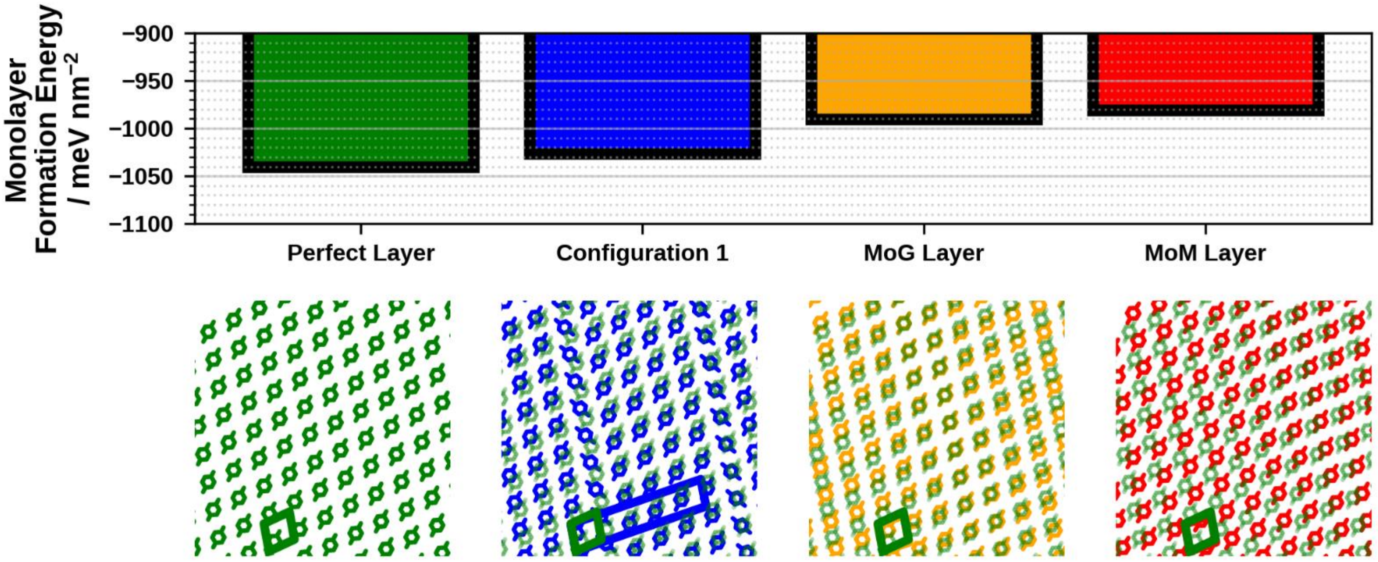

Figure S13. *Comparison of monolayer formation energies for a perfect layer of benzoquinone (see main text), configuration 1 from Figure S11, and the MoG and MoM configuration.*

To gain insight about the stability of configuration 1, we focus on intralayer interactions. To do this, we consider the energy of the configuration 1 layer as a free-standing layer in vacuum. The monolayer formation energy of this configuration is plotted in Figure S13. For comparison, we have the monolayer formation energies of the MoM and MoG configurations, as well as the monolayer formation energy of a benzoquinone perfect layer. With perfect layer we indicate the geometry assumed by a benzoquinone sheet with the same geometry as MoG after a geometry optimization in which the unit cell vectors are allowed to relax, forming the geometry with the best intralayer interactions. We can see in Figure S13 that the intralayer interaction energy of MoG and MoM are extremely similar, with MoG being around 10 meV more stable. This is well predictable, given that the two configurations are almost identical, except for their different alignment on the first layer, and for a slight tilting that MoM molecules adopt to fit on top of the first layer molecules. Configuration 1, on the other hand, is 40 meV more

stable than MoG, and represents a middle point between MoM-MoG and the perfect layer. We can also see that, while the periodicity of MoG and MoG (which is the same as the periodicity of the first molecular layer) is incommensurate to the periodicity of the perfect layer, in the case of configuration 1 the 4 aligned molecules are almost perfectly congruent to the molecules of the perfect layer. In conclusion, we see that the arrangement of the 5 molecules of configuration 1 allows very favorable interlayer interactions, using the elongated 5x1 cell to create stripes of 4 molecules aligned at a very favorable angle, which is different from that of the first molecular layer and is very similar to that of a perfect benzoquinone layer. Therefore, we can conclude that it is a configuration that performs better than MoG and MoM because of its intralayer interactions and not because of a different mechanism of interactions with the previous layer. To understand why this configuration emerges in the case of graphene and not in the case of Ag(111), we must again notice how its cell is an elongated 5x1 cell, in which 5 molecules can be arranged in a row, so that 4 of them align at a very favorable angle, while the 5th molecule closes the gap and allows the structure to fit on the lattice of the first layer. If we now look at the unit cell of the Ag(111) first layer in Figure 1, we can see that it includes two molecules. Therefore, in order to replicate the 5-molecules patter of configuration 1, SAMPLE would need to assemble cells made of 5 primitive unit cells (10 molecules). As indicated in Table S2, this was not the case in our study, because extending the SAMPLE approach to cells of such dimensions on Ag(111) would pose an extremely high computational cost. Therefore, such structures were not generated by SAMPLE in the case of Ag(111).

5. Comparison of models of increasing complexity for reproducing the LUMO-LUMO overlap of different configurations

In Figure 4 the values of LUMO-LUMO overlap for a benzoquinone dimer are shown and used to draw conclusions on the properties of the molecular bilayers found with our structure search method. It is easily noticed that a molecular dimer presents important differences from our real structures, and the suitability of such a dimer model for explaining the variation of overlap between our structures is not to be taken for granted. In particular, it can be noticed that while in Molecule-on-Molecule the top molecule is placed mainly on top of a single bottom-layer molecule, in Molecule-on-Gap the top molecule is placed between two adjacent bottom-layer molecules. As a consequence, a dimer model in which the top molecule can, independently of its position, only interact with one single bottom-layer molecule could be expected to fail in accurately describing the difference between the two configurations. To verify whether this is actually the case, a comparison of LUMO-LUMO overlap has been conducted on a model system in which the top molecules interact with two bottom-layer molecules. In addition, an analogous comparison has been performed on a series of model systems of increasing complexity, including the actual structures of the organic bilayers on graphene and Ag(111) found with our structure search approach. The results of this set of comparisons are shown in Figure S14. It can be observed that the overlap difference detected for the simple dimer model is among the biggest, and passing through more complex systems produces wide variations in the measurements. Overall, the difference always favors MoM. In conclusion, the fact that the magnitude of the difference depends on a variety of factors, with no specific feature dramatically improving the results with respect to the simple dimer model, and that the general trend is preserved, justifies our usage of the dimer model.

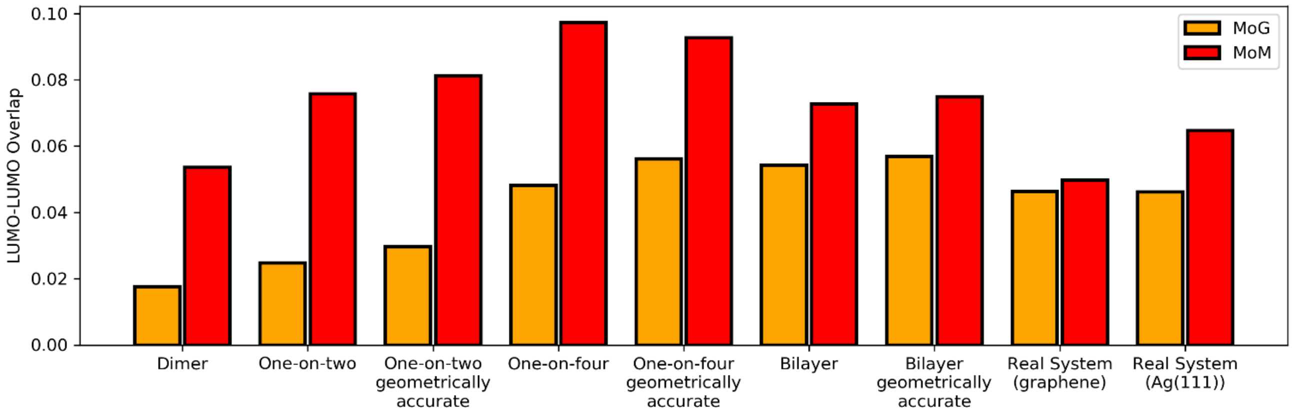

Figure S14. *LUMO-LUMO interlayer overlaps for systems of increasing complexity. Dimer: molecular dimer as presented in Figure 4; one-on-two: cluster with two neighboring molecules in the bottom layer (along the axis of displacement of the top molecule) and one molecule in the top layer; one-on-four: same as one-on-two, with two additional molecules in the bottom layer, at the sides of the first two; bilayer: same as dimer, but under periodic boundary conditions with the same unit cell as in the graphene bilayer; real systems: same structures as obtained with our structure search method, without substrates. "Geometrically accurate" indicates systems in which the intermolecular distance, the tilting of the top molecule and the bonding of the bottom molecule are adjusted to produce an exact replica of the molecules on Ag(111), specifically the strongly bent molecule and the molecule on top of it.*

6. References

(1) Jeindl, A.; Domke, J.; Hörmann, L.; Sojka, F.; Forker, R.; Fritz, T.; Hofmann, O. T. Nonintuitive Surface Self-Assembly of Functionalized Molecules on Ag(111). *ACS Nano* **2021**. https://doi.org/10.1021/acsnano.0c10065.

(2) Wisesa, P.; McGill, K. A.; Mueller, T. Efficient Generation of Generalized {Monkhorst}-{Pack} Grids through the Use of Informatics. *Phys. Rev. B* **2016**, *93* (15), 155109. https://doi.org/10.1103/PhysRevB.93.155109.

(3) Todorović, M.; Gutmann, M. U.; Corander, J.; Rinke, P. Bayesian Inference of Atomistic Structure in Functional Materials. *npj Comput. Mater.* **2019**, *5* (1), 35. https://doi.org/10.1038/s41524-019-0175-2.

(4) Hörmann, L.; Jeindl, A.; Egger, A. T.; Scherbela, M.; Hofmann, O. T. SAMPLE: Surface Structure Search Enabled by Coarse Graining and Statistical Learning. *Comput. Phys. Commun.* **2019**, *244*, 143–155. https://doi.org/10.1016/J.CPC.2019.06.010.

(5) Reuter, K.; Scheffler, M. First-Principles Atomistic Thermodynamics for Oxidation Catalysis: Surface Phase Diagrams and Catalytically Interesting Regions. *Phys. Rev. Lett.* **2003**, *90* (4), 4. https://doi.org/10.1103/PhysRevLett.90.046103.

(6) Ghan, S.; Kunkel, C.; Reuter, K.; Oberhofer, H. Improved Projection-Operator Diabatization Schemes for the Calculation of Electronic Coupling Values. *J. Chem. Theory Comput.* **2020**, *16* (12), 7431–7443. https://doi.org/10.1021/acs.jctc.0c00887.
